# Towards high-throughput screening (HTS) of polyhydroxyalkanoate (PHA) production *via* Fourier transform infrared (FTIR) spectroscopy of *Halomonas* sp. R5-57 and *Pseudomonas* sp. MR4-99

**DOI:** 10.1371/journal.pone.0282623

**Published:** 2023-03-08

**Authors:** Mikkel Christensen, Iulia Chiciudean, Piotr Jablonski, Ana-Maria Tanase, Volha Shapaval, Hilde Hansen

**Affiliations:** 1 Department of Chemistry, UiT The Arctic University of Norway, Tromso, Norway; 2 Department of Genetics, Faculty of Biology, University of Bucharest, Bucharest, Romania; 3 Department of Chemistry, Umeå University, Umeå, Sweden; 4 Faculty of Science and Technology, Norwegian University of Life Sciences, Aas, Norway; Konkuk University, REPUBLIC OF KOREA

## Abstract

High-throughput screening (HTS) methods for characterization of microbial production of polyhydroxyalkanoates (PHA) are currently under investigated, despite the advent of such systems in related fields. In this study, phenotypic microarray by Biolog PM1 screening of *Halomonas* sp. R5-57 and *Pseudomonas* sp. MR4-99 identified 49 and 54 carbon substrates to be metabolized by these bacteria, respectively. Growth on 15 (*Halomonas* sp. R5-57) and 14 (*Pseudomonas* sp. MR4-99) carbon substrates was subsequently characterized in 96-well plates using medium with low nitrogen concentration. Bacterial cells were then harvested and analyzed for putative PHA production using two different Fourier transform infrared spectroscopy (FTIR) systems. The FTIR spectra obtained from both strains contained carbonyl-ester peaks indicative of PHA production. Strain specific differences in the carbonyl-ester peak wavenumber indicated that the PHA side chain configuration differed between the two strains. Confirmation of short chain length PHA (scl-PHA) accumulation in *Halomonas* sp. R5-57 and medium chain length PHA (mcl-PHA) in *Pseudomonas* sp. MR4-99 was done using Gas Chromatography-Flame Ionization Detector (GC-FID) analysis after upscaling to 50 mL cultures supplemented with glycerol and gluconate. The strain specific PHA side chain configurations were also found in FTIR spectra of the 50 mL cultures. This supports the hypothesis that PHA was also produced in the cells cultivated in 96-well plates, and that the HTS approach is suitable for analysis of PHA production in bacteria. However, the carbonyl-ester peaks detected by FTIR are only indicative of PHA production in the small-scale cultures, and appropriate calibration and prediction models based on combining FTIR and GC-FID data needs to be developed and optimized by performing more extensive screenings and multivariate analyses.

## Introduction

Accumulation of PHA polyesters as intracellular inclusions are described in several phyla of bacteria due to *e*.*g*. carbon- and nitrogen availability [1], as protection against various stresses [2], or for release as methyl-esterified oligomeric plant-host effectors [3]. PHA consists of linear chains of alkanoic acid monomers with a stereospecific side chain of variable length depending on supplementation of different types of substrates (*e*.*g*. sugars, alcohols, aromatic compounds, or even- and odd chain length fatty acids), the organism specific flux via metabolic pathways (*e*.*g*. glycolysis, the tricarboxylic acid cycle, fatty acid *de novo* synthesis, or beta-oxidation), and presence of different types of PHA biosynthesis- and synthase enzymes [4]. The alkanoic acid monomers are classified based on the number of carbon atoms (C_x_), with a scl-PHA containing C_4_ to C_5_ and mcl-PHA C_6_ to C_14_ [5].

A recent meta-analysis of the PHA literature found eight dominant research trends in the last 30 years [6], of which manipulation of PHA biosynthesis genes and sustainable production of PHA from waste-based substrates or wastewater treatment plants are considered of high relevance for microbiology research. New PHA producing strains are continuously described [7, 8] and recent advances in genome editing tools have facilitated complex manipulations of PHA production in bacteria such as *E*. *coli*, *Pseudomonas* and *Halomonas* [9, 10]. Despite these current trends resulting in an exponential increase of articles about PHA since the 1990’s [6], only little attention has been paid to the application of HTS approaches for identification of PHA producing strains and optimization of parameters that influence PHA production.

An initial step in screening approaches for finding PHA producing bacteria typically involves staining of intracellular PHA inclusions with fluorescent dyes such as Nile-Red or LipidGreen2 [11, 12]. PHA production is subsequently quantified and characterized by GC-FID or mass spectrometry after freeze drying, solvent extraction, and methanolysis of cells cultivated in liquid cultures [11, 13]. The biomass requirement for GC analyses therefore indirectly leads to use of shake flasks containing milliliter culture volumes. Rapid analysis of microliter scale cultures can therefore increase throughput of screening and optimization experiments, which are currently labor intensive and tedious.

FTIR is highly applicable for identification of putative PHA production in whole- or lyophilized cells obtained directly from agar plates or liquid cultures [14, 15]. Analysis by FTIR generally requires low amounts of cell material and provide spectral information about proteins, carbohydrates, lipids, DNA, and other biomolecules [16]. Although a few studies have established models for quantitating PHA production by FTIR, this technique is usually only considered to be semi-quantitative and to provide relative quantitative information about PHA production [17–19]. The commonly used infrared spectral band for PHA quantification, the carbonyl-ester band, is reported to give a peak absorbance from 1724 cm^-1^ to 1745 cm^-1^ [14, 15, 17, 20]. However, the carbonyl-ester peak is not specific for PHA as other types of lipids such as acylglycerides and rhamnolipids also show signals in this band region [21]. False-positive signals interpreted as PHA are therefore to be expected in FTIR-based PHA screenings similar to the use of lipophilic dyes.

The standardized and commercially available 96-well Biolog phenotype microarray is commonly used for substrate profiling of bacterial strains, to compare closely related type-strains for phenotypic differences, or to assess the potential of various carbon substrates for bio-industrial production [22, 23]. Although the Biolog assay has been used to characterize PHA producing strains in terms of substrate utilization [22, 23], upscaling to a larger culture volume is still required to screen for PHA production. Time- and costs associated with the characterization of PHA phenotypes for newly isolated strains, reference strains in microbial culture collections, or for mutant- and multivariate analyses, can therefore be reduced by developing HTS systems that can measure bacterial production of PHA.

Many species of the Gammaproteobacteria genera *Halomonas* and *Pseudomonas* are producers of PHA with relevance for industrial production [24] and carbon substrate utilization is commonly established as part of a new strain’s description [22, 25–27]. Species of *Halomonas* generally only produce scl-PHA such as polyhydroxybutyrate (PHB) and polyhydroxybutyrate-co-valerate (PHBV), although a recent study indicated production of mcl-PHA in the form of polyhydroxydodecanoate in *Halomonas* sp. 363 [7]. *Pseudomonas* species produce different types of mcl-PHA co-polymers such as polyhydroxyhexanoate (PHH), polyhydroxyoctanoate (PHO), polyhydroxydecanoate (PHD), or special types of PHA such as those containing nitrogen- or sulfur side chain groups [4].

This study aimed to establish a HTS approach for the investigation of PHA production and bacterial growth in microliter scale cultures. For this purpose, two strains from our in-house bacterial collection were selected as potential producers of scl- and mcl-PHA, respectively. PHA metabolism genes have previously been described in the genome of *Halomonas* sp. R5-57 [28], which made it a candidate for production of scl-PHA such as PHB like other described *Halomonadacea* members. Similarly, many *Pseudomonas* species are well-known producers of mcl-PHA, and preliminary studies suggested *Pseudomonas* sp. MR4-99 to be a relevant strain to include in this study. As a first step, Biolog screenings were undertaken to study the metabolic phenotype of 95 carbon substrates for *Halomonas* sp. R5-57 and *Pseudomonas* sp. MR4-99. Growth of the bacteria and screening of PHA production in 96-well plate cultures were subsequently carried out with selected carbon substrates based on the Biolog results. Carbonyl-ester peaks indicative of PHA production were identified in all FTIR spectra from *Halomonas* sp. R5-57 and in most spectra (10 out of 14) obtained from *Pseudomonas* sp. MR4-99. Shifts in the carbonyl-ester peaks were observed between the two strains consistent with production of scl-PHA or mcl-PHA being produced by *Halomonas* and *Pseudomonas*, respectively. To validate the results obtained in small-scale cultures, the results from the growth curve- and FTIR analyses of 96-well plates were finally used to select two substrates for upscaling to 50 mL cultures, from which PHA production was verified by GC-FID analysis. The FTIR analyses of the 50 mL cultures identified carbonyl-ester peaks similar to those in the small-scale cultures, which further supported the hypothesis that PHA was in fact produced in the two experimental systems. To our knowledge, this is the first study that suggest production of PHA in bacteria cultured in 96-well plates and analyzed in a high-throughput manner *via* FTIR. However, although our study showed that FTIR spectroscopy can be used for qualitative screenings of bacterial PHA production, truly quantitative analyses requires development of calibration and prediction models by combining FTIR- and GC-FID data.

## Method

### Media and substrates

Modified lysogeny broth (LB) medium [29] was made with 10 g/L tryptone (Sigma-Aldrich), 5 g/L yeast extract (YE; Merck), and with either 15 g/L NaCl (LB1-5) or 35 g/L NaCl (LB3-5), and autoclaved.

*Pseudomonas* sp. MR4-99 Biolog PM1 liquid medium (ASW_PM1_) consisted of 20 g NaCl, 4.0 g/L Na_2_SO_4_, 0.20 g/L KH_2_PO_4_, 1.4 g/L MgCl_2_, 0.50 g/L KCl, 0.25 g/L NH_4_Cl, 0.112 g/L CaCl_2_, and 0.19 g/L NaHCO_3_, adjusted to pH 7 before autoclaving. After autoclaving and cooling, 10 mL/L of vitamin solution and 1 mL/L of sterile filtered trace element solution were added. Marine peptone (MP) medium was made by adding 1g/L of YE and 5 g/L of tryptone to ASW_PM1_ medium before autoclaving. Agar plates were made by dissolving 15 g/L of agar before autoclaving. Marine agar plates (MA) contained 37.4 g/L Marine Broth (Difco 2216).

*Halomonas* sp. R5-57 Biolog PM1 minimal medium (MM_PM1_) contained 3.0 g/L KH_2_PO_4_, 1.0 g/L NH_4_Cl, 0.19 g/L Na_2_HPO_4_ · 2 H_2_O, 25 g/L NaCl, and adjusted to pH 7 before autoclaving. Sterile filtered solutions of MgSO_4_ (2 mM final) and CaCl_2_ (1 mM final) were added to MM_PM1_ medium after autoclaving and cooling.

PHA production medium (MM_PHA1_) used for *Halomonas* sp. R5-57 consisted of 3.0 g/L KH_2_PO_4,_ 0.20 g NH_4_Cl, 0.19 g/L Na_2_HPO_4_ · 2 H_2_O, and 35 g/L NaCl. Separately autoclaved NaHCO_3_ (4.0 g/L final) was added with sterile filtered solutions of MgSO_4_ (2.0 mM final), CaCl_2_ (1.0 mM final), and vitamins and trace elements solutions were added as previously stated to the MM_PHA1_. The PHA production medium (MM_PHA2_) for *Pseudomonas* sp. MR4-99 was prepared in the same manner using 15 g/L of NaCl and by substituting NH_4_Cl with 1.0 g/L of YE (Merck).

The vitamin solution contained 0.2 g/L biotin (Sigma-Aldrich), 2.0 g/L nicotinic acid (VWR), and 0.8 g/L 4-aminobenzoic acid (Sigma-Aldrich) [30]. The trace element solution contained 2.1 g/L Fe(SO_4_) · 7H_2_O, 13 mL/L 25% HCl, 5.2 g/L Na_2_EDTA · 2 H_2_O, 30 mg/L H_3_BO_3_, 0.1 g/L MnCl_2_ · 4H_2_O, 0.19 g/L CoCl_2_ · 6H_2_O, 2 mg/L CuCl_2_ · 2H_2_O, 0.144 g/L ZnSO_4_ · 7 H_2_O, and 36 mg/L Na_2_MoO_4_ ·2 H_2_O [30].

Sterile flat-bottom 96-well plates were prepared fresh with substrates supplied in concentrations shown in Table 1. The concentrations were calculated based on achieving carbon to nitrogen ratios of 24.2 (± 2.1) for *Halomonas* sp. R5-57 or 12.1 (± 1.0) for *Pseudomonas* sp. MR4-99 by adjusting for the assumed number of acetyl-CoA equivalents generated per molecule of substrate (Table 1). Stock solutions of propionate (Merck) and valerate (Alfa Aesar) were neutralized to pH 7 using sodium hydroxide, sterilized by filtration using a 0.2 μm PES syringe filter (VWR) and supplied to 0.06 M or 0.09 M (final), respectively. The assumed acetyl-CoA equivalents were based on reaction pathways obtained from Kyoto Encyclopedia of Genes and Genomes (KEGG, www.kegg.jp/kegg/pathway.html#metabolism).

**Table 1:** List of carbon substrates.

Substrate	Acetyl-CoA equiv.	Concentration (g/L)	Manufacturer
Sucrose	4	8.0	VWR
L-Arabinose	2	7.0	Sigma
D-Galactose	2	8.5	AppliChem
D-Mannitol	2	8.5	Sigma
D-Fructose	2	8.5	VWR
a-D-Glucose	2	8.5	VWR
D-L-Malic acid	1	12.5	Sigma
Na-gluconate	2	9.1	VWR
Xylose	2	6.9	Merck
L-Lactic acid	1	8.4	Honeywell
Na-acetate	1	5.6	VWR
Mannose	2	8.4	VWR
*N*-acetylglucosamine	2	10.3	Carbosynth
Ethanol	1	4.5	VWR
Glycerol	1	8.6	VWR

Substrates used in 96-well growth assays, and their assumed acetyl-CoA equivalents used to calculate the concentrations supplied in the PHA production medium.

### Bacterial strains and conditions for starter cultures

*Halomonas* sp. R5-57 and *Pseudomonas* sp. MR4-99 were isolated from the Barents sea and obtained from Marbank—The National Marine Biobank of Norway (Tromsø, Norway) [28]. Both strains were streaked on MA plates from cryo-stocks (- 80°C, 20% glycerol) and incubated for 1 day at room temperature (~ 25°C). Individual colonies were picked and, unless otherwise stated, inoculated into 3 mL of LB1-5 (*Pseudomonas* sp. MR4-99) or LB3-5 (*Halomonas* sp. R5-57) medium for preparing starter cultures in biological triplicates and incubated with 1270 shakes per minute on a Heidolph Multi Reax multi-vortexer at room temperature. Overnight cultures were re-suspended into fresh medium to an optical density at 600 nm (OD_600nm_) of ~ 0.15 and incubated until early- to middle exponential phase (OD_600nm_ of 0.6–1.5). Similarly, overnight starter cultures were used to inoculate 50 mL LB1-5 or LB3-5 medium and incubated until OD ~ 1 for use in shake flask PHA production experiments.

### Biolog screening

A single colony of *Pseudomonas* sp. MR4-99 grown on MP agar was inoculated in 100 mL MP medium and cultivated for 24 hours at 20°C with 150 rpm. The cells were separated from the supernatant by centrifugation at 5000 rpm for 10 minutes, and washed 2 times with ASW_PM1_ medium. Then the cell culture was diluted with the same medium to a start inoculum transmittance of 50% measured on a Biolog Turbidimeter (Biolog, USA) according to the manufacturer’s instruction. *Halomonas* sp. R5-57 was cultivated and washed similarly, but in MM_PM1_ medium.

Biolog PM1 plates (Biolog, USA) were inoculated with a volume of 150 μL per well of strain specific medium (50% transmittance) and incubated in sealed plastic bags without shaking at 20°C for 216 hours (*Pseudomonas* sp. MR4-99) or at 25°C for 144 hours (*Halomonas* sp. R5-57). The tetrazolium dye reduction was measured in a MicroStation ELX808BLG Reader Analyser (Biolog, USA) at 590 nm. The average absorbance values were calculated from three independent experiments consisting of one Biolog plate each for *Halomonas* sp. R5-57. The background absorbance from the *“no carbon”* control well was subtracted from each measurement using a Python script developed for the plate reader specific output files available at https://github.com/bast/mikkel-wells. Data from *Pseudomonas* sp. MR4-99 were obtained from a single experiment consisting of one Biolog PM1 plate.

### Growth assays in 96-well plates

Cells from starter cultures were separated by centrifugation for 3 minutes at 8000 *g* in sterile Eppendorf tubes, and washed and re-suspended in 10 mL MM_PHA1_ (*Halomonas* sp. R5-57) or MM_PHA2_ (*Pseudomonas* sp. MR4-99). Next, 150 μL of the re-suspended cells were transferred to a 96-well plate prepared with fresh carbon substrates (Table 1) and sealed with transparent Breathe-Easy® polyurethane sealing membrane (Sigma-Aldrich). The plates were then incubated for 72 hours (*Pseudomonas* sp. MR4-99) or 96 hours (*Halomonas* sp. R5-57) in a Synergy H1 plate reader with the temperature set to 25°C and the double orbital continuous shaking frequency set to 425 cpm (3 mm). The OD_600nm_ was measured every 15 minutes. Each individual experiment consisted of one 96-well plate.

Growth curve metrics were calculated using the “*fit*” function in AMiGA [31] run in Python v. 3. 8. 12 from each experiment with three wells per condition (carbon substrate) corresponding to three biological replicates. Outliers from the growth curve modeling were detected by box plots of the AMiGA output metrics “*growth rate* (gr)”, *time point at which maximum growth rate is reached* (t_gr)”, the “*mean squared error* (MSE)”, and “*area under the curve* (AUC)”. The *Pseudomonas* sp. MR4-99 dataset consisted of two 96-well plates where 10 outliers (wells) were removed. The *Halomonas* sp. R5-57 dataset consisted of four 96-well plates where 27 outliers (wells) were removed.

### Shake flask PHA production

Cells from starter cultures were spun down in sterile 50 mL tubes at 11000 *g* for 10 minutes at 25°C and re-suspended in 50 mL MM_PHA1_ (*Halomonas* sp. R5-57) or MM_PHA2_ (*Pseudomonas* sp. MR4-99) medium in 250 mL baffled flasks incubated at 25°C, 200 rpm for 120 hours (*Halomonas* sp. R5-57) or 72 hours (*Pseudomonas* sp. MR4-99). Samples for measuring OD_600nm_ (100 μL) and for FTIR analysis (900 μL) were taken at 24 hours interval.

At the end of the experiment, 40 mL of culture was centrifuged in pre-weighed 50 mL plastic tubes at 18000 *g*, 20 minutes at 4°C and washed one time with 15 g/L (*Pseudomonas* sp. MR4-99) or 35 g/L (*Halomonas* sp. R5-57) sodium chloride solution. The bacterial pellets were frozen at—80°C before lyophilization, analytical weighing, and GC-FID analysis for identification and quantification of PHA production.

### FTIR analysis

Infrared spectra were obtained from bacterial samples by two FTIR spectroscopy systems, a Cary 530 (Cary-FTIR) Attenuated Total Reflectance (ATR)-FTIR (Agilent, USA) and a High Throughput Screening eXTension (HTS-XT) unit coupled to the Vertex 70 FTIR spectrometer (Bruker Optik, Germany).

### Cary-FTIR analysis

Bacterial cells were pooled from three replicate wells (~ 450 μL in total) and centrifuged 2 minutes at 21000 *g*. The cell pellet was then washed with 96% ethanol, centrifuged again and applied to the FTIR sensor using a steel micro spatula. Two 96-well plates from two independent experiments per strain were analyzed. Samples obtained from 50 mL cultures (900 μL) to be analyzed on the Cary-FTIR system were centrifuged, washed as described above and analyzed in technical triplicates. The Cary-FTIR spectra were collected in the range 650–4000 cm^-1^ with 64 background scans and 256 sample scans using HappGenzel apodization without zero fill factor using Agilent MicroLab PC software.

### Bruker HTS-XT FTIR analysis

The bacterial cultures (~ 150 μL each) from a single experiment for each strain consisting of one 96-well plate were transferred into 96-well PCR reaction tubes and centrifuged at 7600 rpm for 15 minutes at 4°C. The cell pellets were then washed with 100 μL 96% ethanol (VWR) after removal of the supernatant, centrifuged again and the supernatant was removed. The cells were then re-suspended and mixed in approx. 5 μL ethanol (dependent on the pellet size) and then applied onto the IR-light-transparent 384-well FTIR plate by pipetting approximately 1 μL of the suspension in technical triplicates, with an empty well used as spacing between samples from different experimental conditions. The HTS-XT FTIR spectra were collected in the range 400–4000 cm^-1^ with 64 background and sample scans at a resolution of 6 cm^-1^ using Brukers Opus software.

Spectra from both FTIR systems were normalized to an absorbance of 0.5 at 1650 cm^-1^, baseline corrected using the linear function, and the carbonyl-ester and amide band I-ester regions calculated with the *“Individual baseline”* integral function in SpectraGryph, Software for optical spectroscopy v. 1.2.15 (Dr. Friedrich Menges, Oberstdorf, Germany). The carbonyl- and amide band I-ester regions were defined as 1763–1705 cm^-1^ and 1705–1580 cm^-1^ for Cary-FTIR spectra, and 1772–1710 cm^-1^ and 1710–1600 cm^-1^ for HTS-XT-FTIR spectra.

### Quantification of PHA by GC-FID

The amount of PHA in the bacterial biomass was quantified by GC-FID after methanolysis as described previously [32], except that a methanolysis reaction time of 2 hours and a phase separation volume of 0.75 mL of Milli-Q water were used. Methyl-3-hydroxybutyrate (Sigma-Aldrich), methyl-3-hydroxyhexanoate (Sigma-Aldrich), methyl-3-hydroxyoctanoate (Larodan-AB), and methyl-3-hydroxy-decanoate (Larodan-AB) were used as external standards. PHA quantification is reported as the percentage of cell dry weight (CDW) of the freeze dried bacterial pellets.

### Statistical analysis

Results reported as average values ± standard deviation were calculated using the functions “groupby.mean” and “groupby.std” in Python Pandas v. 1.1.3 run in Python v. 3.8.8. The box plots (linear method), heat maps, bar graphs, and scatter plots were coded with Python Plotly v. 5.6.0 run in Python v. 3.8.8.

## Results

**Fig 1:**
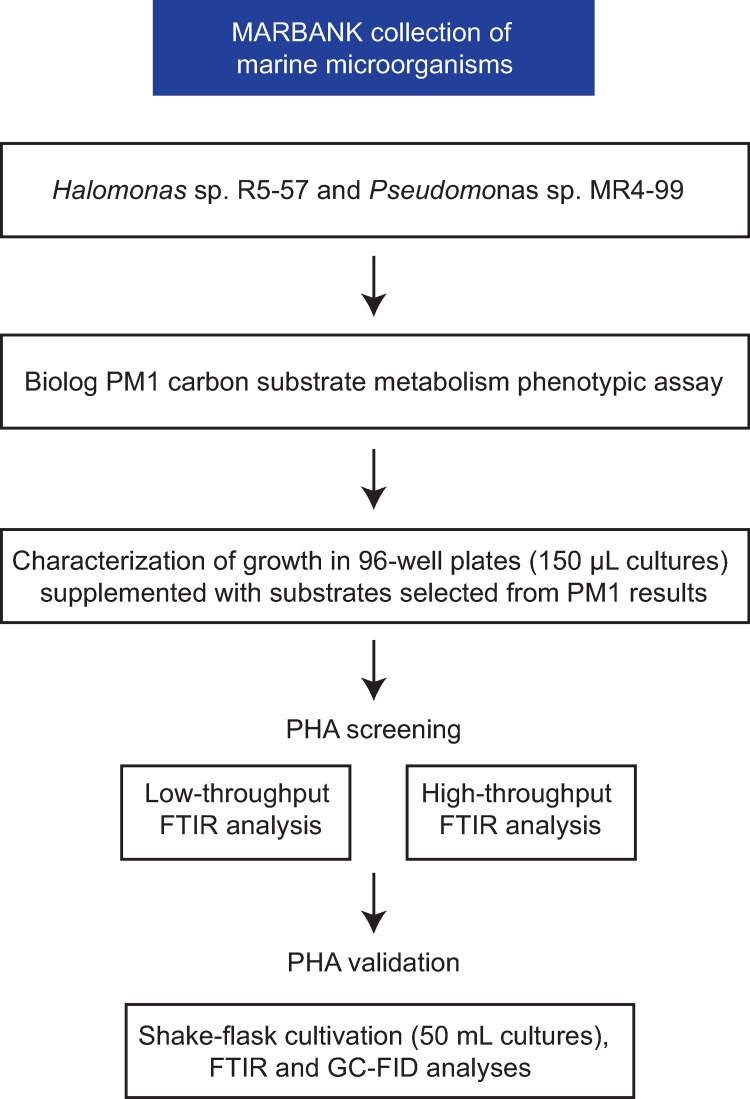
Overview of the experimental workflow in this study.

Increasing the throughput in screening- and optimization of bacterial PHA production is desirable in order to efficiently characterize wild type isolates deposited in culture collections and mutants from targeted libraries, or to evaluate the influence of cultivation parameters such as carbon or nitrogen substrates, temperature, salt or medium composition. As outlined in Fig 1, the aim of this study was to evaluate a HTS system for analysis of bacterial growth and PHA production.

Two bacterial strains from the Marbank collection (Tromsø, Norway) were selected based on preliminary experiments suggesting PHA production (*Pseudomonas* sp. MR4-99) and the presence of PHA biosynthesis genes in the annotated genome of *Halomonas* sp. R5-57 [28]. The strains have not been well characterized in terms of growth requirements nor PHA production, so metabolic phenotypes from 95 carbon substrates were initially generated using Biolog PM1 plates. Some of the carbon substrates that gave highest metabolic activity as measured by changes in absorbance (Abs_590nm_) were subsequently selected for characterization of growth in 96-well plates and screened for putative PHA production *via* FTIR analysis on two different FTIR systems. Finally, two promising substrates were used as supplement in 50 mL cultures and analyzed by FTIR and GC-FID to verify PHA production in both strains.

### Carbon substrate profiling using Biolog PM1

The Biolog assay measures changes in absorbance at 590 nm (Abs_590nm_) due to reduction of a tetrazolium dye to formazan by electrons donated from nicotinamide adenine dinucleotide (NADH), which is generated from oxidation of the substrates solubilized in individual wells in a 96-well plate [33, 34]. Here, Biolog absorbance measurements (Abs_590nm_) were converted into heat maps to show how the substrates were metabolized over time. Some of the 95 substrates generated a much stronger metabolic response than others (S1 and S2 Figs). In total, 49 substrates were metabolized by *Halomonas* sp. R5-57 and 54 substrates by *Pseudomonas* sp. MR4-99, as defined by exceeding an arbitrary threshold absorbance value of 0.15 above the *“No carbon”* control well (low absorbance cut-off). When setting the cut-off values at 0.30 (medium absorbance cut-off) or 1.00 (high absorbance cut-off), *Halomonas* sp. R5-57 metabolized 44 or 25 substrates, while *Pseudomonas* sp. MR4-99 metabolized 51 or 20 substrates.

Substrates that exceeded the medium- or high absorbance cut-off values in the Biolog PM1 assay after 24, 48, 96, or 144 hours, were considered of interest to be characterized for growth curve analysis and screening of PHA production. Substrates such as amino acids whose metabolism generally do not result in PHA production were excluded from the PHA screening.

**Fig 2:**
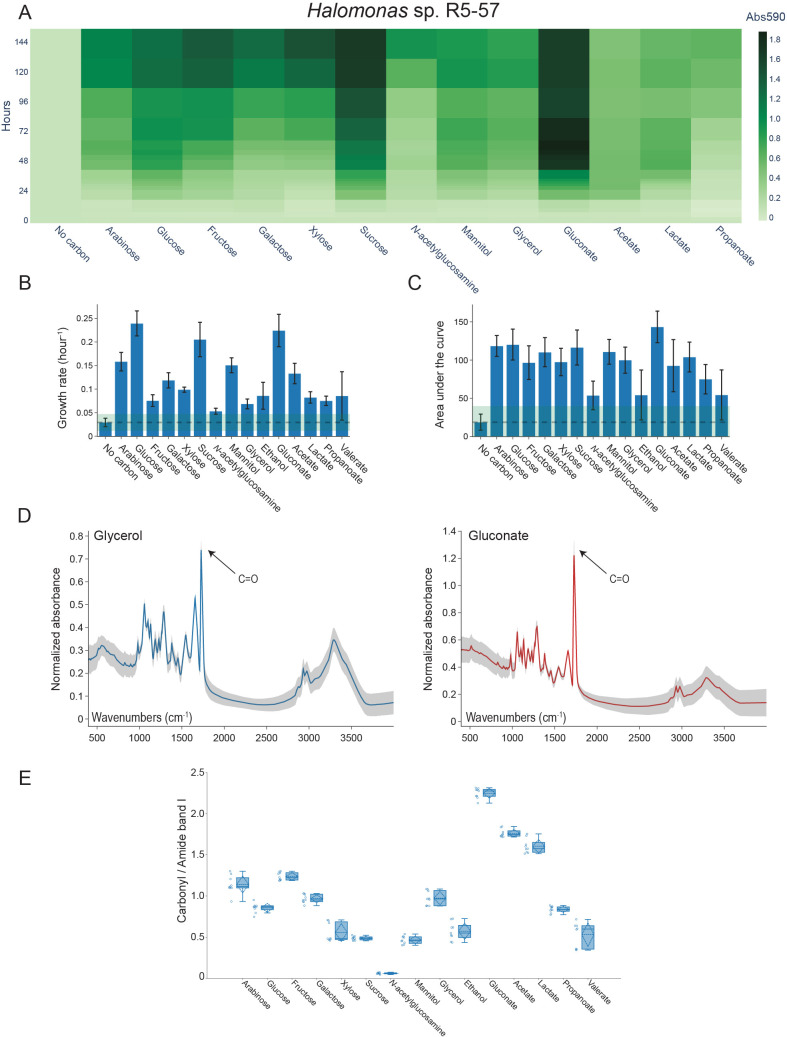
High-throughput PHA screening of *Halomonas* sp. R5-57 cultivated in 96-well plates. (A) Heat map showing the Biolog PM1 substrate utilization measured as absorbance at 590 nm (Abs_590nm_) for 13 selected substrates (n = 3). (B) Bar graph depicting the growth rate (“maximum specific growth rate”) in MM_PHA1_ medium supplemented with the indicated carbon substrates (n = 4). The growth rate (stippled line) is shown for the “No carbon” control plus/minus two standard deviations (green box). (C) Bar graph depicting the average AUC for 15 carbon substrates (n = 4) and for the “No carbon” control (stippled line) plus/minus two standard deviations (green box). (D) Normalized FTIR spectra obtained from cells supplemented with glycerol or gluconate showed a carbonyl-ester peak at wavenumber 1728 (± 2) cm^-1^, which indicated production of PHB (n = 3 and analyzed with 3 technical replicates on a Bruker HTS-XT FTIR). The standard deviation is shown as shaded areas. (E) Box plot showing the ratio of the carbonyl-ester to amide-ester calculated from FTIR spectra similar to shown in Fig 2D. Open dots represent individual measurements, while the box contains the mean value (stippled horizontal lines) and standard deviation (stippled vertical lines), the median value (full horizontal line), the upper and lower fences, and quartile 1 and 3 (box edges).

**Fig 3:**
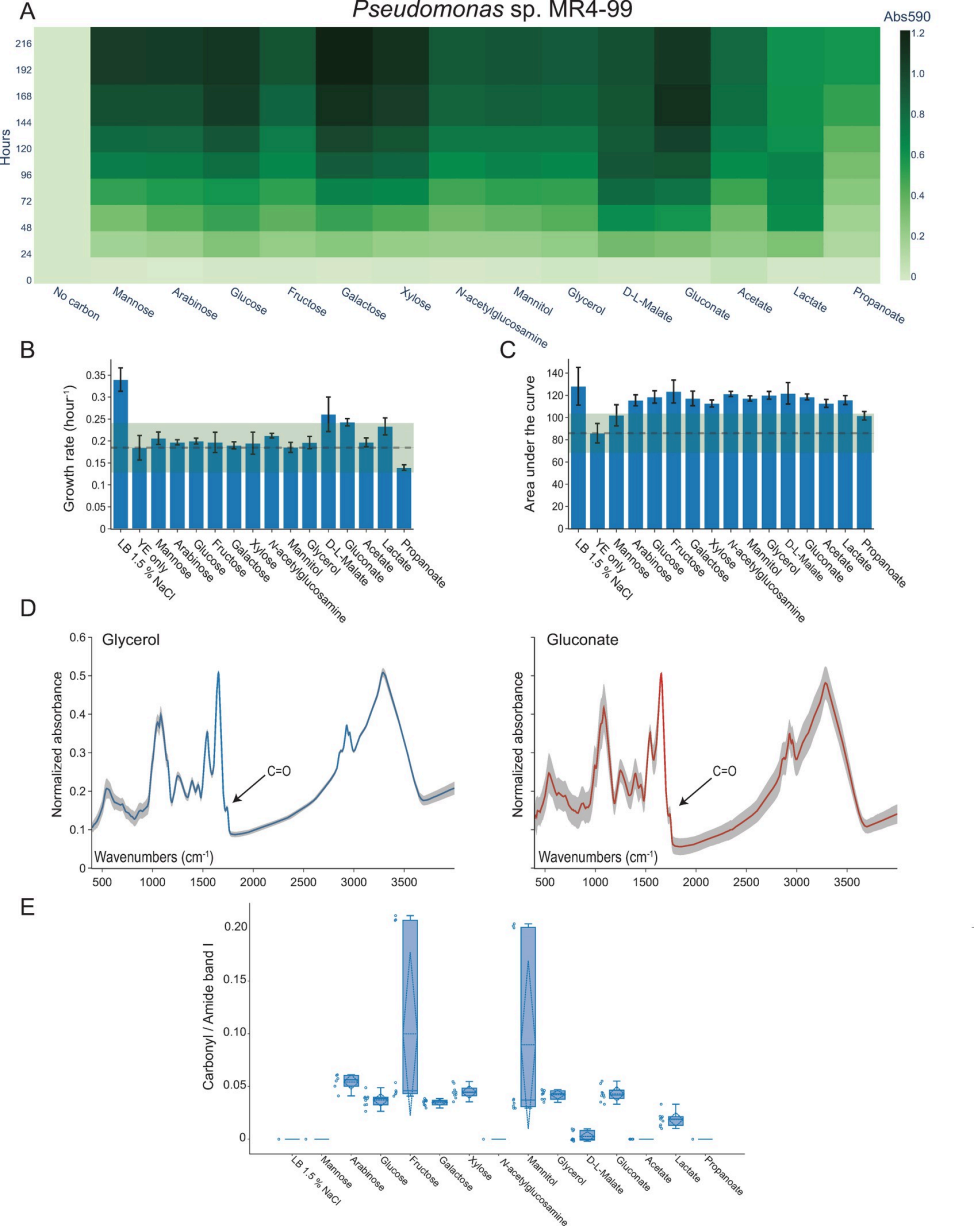
High-throughput PHA screening of *Pseudomonas* sp. MR4-99 cultivated in 96-well plates. (A) Heat map showing the Biolog PM1 substrate utilization measured as absorbance at 590 nm (Abs_590nm_) for 14 selected substrates (n = 3). (B) Bar graph depicting the growth rate (“maximum specific growth rate”) in MM_PHA2_ medium supplemented with the indicated carbon substrates (n = 2). The growth rate (stippled line) is shown for the “YE only” control plus/minus two standard deviations (green box). (C) Bar graph depicting the average AUC for 14 carbon substrates (n = 2) and for the “No carbon” control (stippled line) plus/minus two standard deviations (green box). (D) Normalized FTIR spectra obtained from cells supplemented with glycerol or gluconate showed a carbonyl-ester peak at wavenumber 1738 (± 3) cm^-1^, which indicated production of mcl-PHA (n = 3 and analyzed with 3 technical replicates on a Bruker HTS-XT FTIR). The standard deviation is shown as shaded areas. (E) Box plot showing the ratio of the carbonyl-ester to amide-ester calculated from FTIR spectra similar to shown in Fig 3D. Open dots represent individual measurements, while the box contains the mean value (stippled horizontal lines) and standard deviation (stippled vertical lines), the median value (full horizontal line), the upper and lower fences, and quartile 1 and 3 (box edges).

The highest absorbance values in the Biolog PM1 assay for *Halomonas* sp. R5-57 were found for gluconate, sucrose, xylose, fructose, glucose, and galactose, as depicted in the heat map in Fig 2A with the 13 carbon substrates (out of 15) included in the PHA production screening. For *Pseudomonas* sp. MR4-99, the similar plot provided in Fig 3A revealed gluconate, galactose, xylose, glucose, arabinose, and mannose to exceed the high absorbance cut-off value, out of 14 substrates selected for subsequent PHA production screening.

### Growth curve analysis of *Halomonas* sp. R5-57 and *Pseudomonas* sp. MR4-99 cultured in 96-well plates

In order to characterize growth and putative PHA production in a HTS setup, 96-well plates were supplemented with strain specific medium and a single carbon substrate in each individual well. The selection of carbon substrates was based on results from the Biolog assay (Figs 2A and 3A) with additional inclusion of ethanol and valerate as substrates for *Halomonas* sp. R5-57, due to their potential relevance for industrial production of PHA such as PHBV with high valerate content [35, 36]. Therefore, a total of 15 substrates were chosen for growth curve analysis and screening of PHA production for *Halomonas* sp. R5-57 and 14 substrates for *Pseudomonas* sp. MR4-99.

The carbon substrate concentrations used were based on the calculated number of acetyl-CoA equivalents generated from each molecule of substrate, obtained from KEGG Pathway Maps [37–39]. Preliminary experiments using *Pseudomonas* sp. MR4-99 revealed that replacement of ammonium chloride with YE provided better growth and higher putative PHA production which resulted in use of a lower carbon to nitrogen (C:N) ratio compared to *Halomonas* sp. R5-57, although the same absolute substrate concentrations were used for both strains. The incubation time in the 96-well plates for the two strains also differed slightly with 72 hours for *Pseudomonas* sp. MR4-99 versus 96 hours for *Halomonas* sp. R5-57. The OD_600nm_ was monitored continuously at 15 minutes intervals, until the end of the experiment where the cells were harvested and analyzed for PHA production *via* FTIR analysis.

As shown in Fig 2B and 2C for *Halomonas* sp. R5-57 and in Fig 3B and 3C for *Pseudomonas* sp. MR4-99, two metrics calculated by the recently published Python script AMiGA were chosen to evaluate growth of the strains when fed with 15 or 14 different carbon substrates: the “maximum specific growth rate” (growth rate) and the AUC [31]. The growth rate corresponds to the point on the growth curve where fastest growth is observed, while the AUC describes the growth curve over the entire time interval and thus incorporate contributions from the initial population size, maximum specific growth rate, and the carrying capacity, into a single metric [40]. As shown in Fig 2B for *Halomonas* sp. R5-57, the growth rate was significantly higher than the “No carbon” control for all of the substrates tested (*p* < .05). Glucose, gluconate, and sucrose showed the highest growth rates with values above 0.20 hour^-1^ and arabinose and mannitol above 0.15 hour^-1^. Several substrates showed significantly lower growth rates (*p* < .05) and the substrates fructose, *N*-acetylglucosamine, glycerol, ethanol, lactate, propanoate and valerate showed growth rates below 0.10 hour^-1^. However, when inspecting the AUC metric that describes the full growth curve, the differences between the substrates were less pronounced (Fig 2C). Gluconate, glucose, sucrose and arabinose showed highest AUC (143 ± 20.7, 120 ± 20.3, 116 ± 22.9, and 118 ± 13.7, respectively) but the standard deviations were relatively high. The analyses also show that fructose, galactose, xylose, mannitol, glycerol, acetate, and lactate supported good growth of *Halomonas* sp. R5-57.

For *Pseudomonas* sp. MR4-99, only LB medium showed significantly higher growth rate than the “YE only” control (*p* < .05) (Fig 3B). This is likely due to the presence of growth promoting nitrogen and carbon in the YE. The highest growth rates for *Pseudomonas* sp. MR4-99 were found for D-L-malate, gluconate, and lactate (0.26 ± 0.04 hour^-1^, 0.24 ± 0.01 hour^-1^, 0.23 ± 0.02 hour^-1^). The calculated AUC values for *Pseudomonas* sp. MR4-99 (Fig 3C) showed that all substrates except mannose and propanoate gave significantly higher AUCs than the “YE only” control (*p* < .05). No significant differences in AUC values between the tested substrates (except for mannose and propanoate) could be observed for *Pseudomonas* sp. MR4-99 (*p* < .05). Thus, arabinose, glucose, fructose, galactose, xylose, *N*-acetylglucosamine, mannitol, glycerol, D-L-malate, gluconate, acetate, and lactate are all considered good substrates for growth of *Pseudomonas* sp. MR4-99.

### FTIR analysis of *Halomonas* sp. R5-57 and *Pseudomonas* sp. MR4-99 cultivated in 96-well plates indicated production of scl-PHA and mcl-PHA, respectively

FTIR is a well-established method for analysis of lipids including PHA in bacterial cells [16]. The carbonyl-ester peak of mcl-PHAs is typically observed at a higher wavenumber than scl-PHA, and the FTIR spectra can therefore also be used to distinguish the type of PHA produced [14]. The exact carbonyl-ester peak position depends on the crystallinity of the PHA, which is primarily a function of the PHA side chain configuration but also dependent on other factors such as the water content in the sample and the type of FTIR instrument in use [20].

In this study, the washed cells of *Halomonas* sp. R5-57 and *Pseudomonas* sp. MR4-99 cultured in 150 μL strain specific medium in 96-well plates were analyzed in a low- and a high-throughput FTIR system. As a low-throughput FTIR system, we used the Cary 530 ATR-FTIR (Cary-FTIR), which required pooling the cells from three biological replicates (3 × 150 μL) in order to obtain enough biomass for one measurement. The high-throughput system was the HTS-XT FTIR (Bruker Optik, Germany) which allowed HTS capability of up to 180 cell suspensions in a single run, obtained from individual wells of the 96-well plate with up to three technical replicates. While the high-throughput system is advantageous in terms of increased statistical power, reduced biomass requirement and reduced analysis time per sample, it is expensive equipment compared to *e*.*g*. the Cary-FTIR. Thus, the Cary-FTIR was included in this study to test if cheaper and more commonly available FTIR instruments can be used to analyze bacterial cells cultivated in 96-well plates for putative production of PHA.

Analyses of HTS-XT FTIR spectra obtained from the two strains cultured in 96-well plates showed carbonyl peaks at strain specific peak wavenumbers shown in Fig 2D for *Halomonas* sp. R5-57 and Fig 3D for *Pseudomonas* sp. MR4-99. The strain specific peak wavenumbers for the carbonyl-ester peaks were identified at 1728 (± 2) cm^-1^ for *Halomonas* sp. R5-57 or at 1738 (± 3) cm^-1^ for *Pseudomonas* sp. MR4-99. The peak wavenumbers were slightly different when similar samples were analyzed on the Cary-FTIR, but the strain specific differences remained the same and the carbonyl-ester peaks centered at 1723 ± 2 cm^-1^ for *Halomonas* sp. R5-57 or at 1738 ± 3 cm^-1^ for *Pseudomonas* sp. MR4-99 (S3 and S4 Figs). A carbonyl-ester peak at 1723 ± 2 cm^-1^ is similar to the peak wavelength for PHB verified by GC-FID as previously reported by us for two closely related *Cobetia* spp. strains using the same FTIR instrument [32]. Thus, a higher wavelength of the carbonyl-ester peak identified in *Pseudomonas* sp. MR4-99 spectra suggests production of mcl-PHA.

To quantitatively compare the putative PHA production after supplement with different carbon substrates, the normalized HTS-XT FTIR spectra were converted into carbonyl- to amide band I-ester (CA1) ratios. The carbonyl peak in the FTIR spectra indicates PHA and the amide band I reflects the total protein content of the cell. The amide band I can therefore be used as an internal standard for normalization of spectra [20] and the ratio between the two for quantification of PHA [18].

Carbonyl-ester peaks were observed in the HTS-XT FTIR spectra obtained from the 15 tested carbon substrates for *Halomonas* sp. R5-57 (S5 Fig). As shown in Fig 2E, gluconate showed the highest CA1 ratio exceeding 2.2. Acetate, lactate, galactose, arabinose, and glycerol were all good carbon substrates as well, with CA1 ratios exceeding 1.0. Supplementing the cultures with xylose, sucrose, mannitol, ethanol, propanoate, or valerate resulted in CA1 ratios above 0.50, and these substrates are therefore also potentially of interest for use in PHA production in *Halomonas* sp. R5-57. Although a very low CA1 ratio (0.06 ± 0.01) was observed when *N*-acetylglucosamine was used (Fig 2E), inspection of the normalized Cary- and HTS-XT FTIR spectra showed presence of a carbonyl-ester peak in both (S3 and S5 Figs).

As shown in Fig 3E, the highest CA1 ratios (0.10 ± 0.08 and 0.09 ± 0.08) were found when *Pseudomonas* sp. MR4-99 was supplemented with fructose and mannitol, although only 10 of the tested substrates showed CA1 ratios above zero. Closer inspection of the box plot (Fig 3E) revealed that the high CA1 values for fructose and mannitol were both heavily biased by a single biological replicate analyzed in technical triplicates, which were significantly higher than the other two biological replicates originating from individual wells in the 96-well plate. Thus, the median CA1 values for fructose and mannitol were in the same range as arabinose, glucose, galactose, xylose, glycerol and gluconate (0.05 ± 0.01). Taken together, the median and average CA1 values close to 0.05 indicated relatively low putative PHA production. However, inspection of the normalized HTS-XT FTIR spectra clearly showed true carbonyl-ester peaks, except for ambiguous spectra obtained from *Pseudomonas* sp. MR4-99 supplemented with D-L-malate (S6 Fig). This suggests that mcl-PHA could be produced from 10 out of the 14 substrates supplemented to *Pseudomonas* sp. MR4-99 when cultivated in 96-well plates (150 μL cultures).

### Comparing low-throughput versus high-throughput FTIR

Some discrepancies were observed between the low-throughput Cary-FTIR and high-throughput HTS-XT FTIR analyses of *Halomonas* sp. R5-57. The highest CA1 ratio on both FTIR systems was found from cells supplemented with gluconate (Fig 2E and S7 Fig). Except *N*-acetylglucosamine, all other conditions tested showed higher CA1 ratio in the HTS-XT FTIR dataset, and the CA1 ratio from *Halomonas* sp. R5-57 supplemented with gluconate was almost four times higher than the samples analyzed on the Cary-FTIR instrument.

For *Pseudomonas* sp. MR4-99, the Cary-FTIR data showed glycerol to give the highest CA1 ratios (S8 Fig). However, in comparison, the similar cultured cells supplemented with glycerol but analyzed on the HTS-XT FTIR showed approximately 7-fold lower CA1 ratio. Likewise, Cary-FTIR analyses of *Pseudomonas* sp. MR4-99 cells grown in medium supplemented with mannitol and gluconate also showed higher CA1 ratios than the similar samples analyzed on the HTS-XT FTIR system. *Pseudomonas* sp. MR4-99 cells supplemented with lactate showed a positive CA1 ratio only in the HTS-XT FTIR analysis which was confirmed by inspection of the normalized spectra (S4 and S6 Figs). In contrast, *Pseudomonas* sp. MR4-99 cells supplemented with propanoate showed a carbonyl-ester peak only in the Cary-FTIR analysis (S4 and S6 Figs). Thus, some ambiguities between the FTIR analyses were found.

It should be pointed out that the samples analyzed by the two FTIR systems were from independent experiments, where both biological and technical variation is expected to influence the results.

### Production of scl-PHA in *Halomonas* sp. R5-57 and mcl-PHA in *Pseudomonas* sp. MR4-99

As shown in the previous section, the FTIR analyses revealed carbonyl-ester peaks indicative of PHA production from several carbon sources in *Halomonas* sp. R5-57 and *Pseudomonas* sp. MR4-99 cultivated in 96-well plates. To verify that the carbonyl-ester peaks observed at strain specific wavenumbers originated due to PHA production, the cultures were scaled up to 50 mL and analyzed by GC-FID after freeze-drying and methanolysis reaction. Glycerol and gluconate were chosen as carbon substrates since they provided good growth for both bacterial strains and resulted in clearly visible carbonyl-ester peaks in the spectra obtained from the two FTIR systems.

As shown in Table 2, the GC-FID analysis of *Halomonas* sp. R5-57 verified that PHA was produced when glycerol or gluconate was supplemented with production of 15–17% of the homopolymer PHB from both substrates. For *Pseudomonas* sp. MR4-99, mcl-PHA production of heteropolymers consisting of PHH, PHO, and PHD were found in the range of 41–44% PHA. However, a low CDW was obtained with *Pseudomonas* sp. MR4-99, which led to almost similar PHA volumetric production as demonstrated for *Halomonas* sp. R5-57. The majority of the mcl-PHA polymer produced by *Pseudomonas* sp. MR4-99 consisted of PHO and PHD with only a small percentage of PHH, in addition to trace amounts of scl-PHA in the form of PHB (Table 2).

**Table 2:** PHA production characteristics.

	*Halomonas* sp. R5-57		*Pseudomonas* sp. MR4-99	
	Glycerol	Gluconate	Glycerol	Gluconate
PHB (%)	17.0 ± 1.24	15.6 ± 3.16	1.42 ± 0.32	1.04 ± 0.58
PHH (%)	-	-	2.84 ± 1.27	2.56 ± 0.73
PHO (%)	-	-	17.2 ± 6.08	14.2 ± 3.13
PHD (%)	-	-	22.5 ± 10.8	23.6 ± 5.00
PHA (%), total	17.0 ± 1.24	15.6 ± 3.16	44.0 ± 17.8	41.4 ± 9.43
CDW (g/L)	3.9 ± 0.6	4.2 ± 0.4	1.2 ± 0.4	1.1 ± 0.1
PHA production (g/L)	0.59 ± 0.03	0.57 ± 0.04	0.55 ± 0.38	0.47 ± 0.17
CA1 ratio (AU)	0.99 ± 0.02	1.02 ± 0.01	0.42 ± 0.29	0.40 ± 0.11

PHA production is reported as weight percentages (%) of CDW in 50 mL shake flask cultures as determined by GC-FID analysis and FTIR analysis (± s.d calculated between two individual experiments each with three biological replicates and three technical replicates for the FTIR analysis). CA1 ratios are reported as arbitrary units (AU).

**Fig 4:**
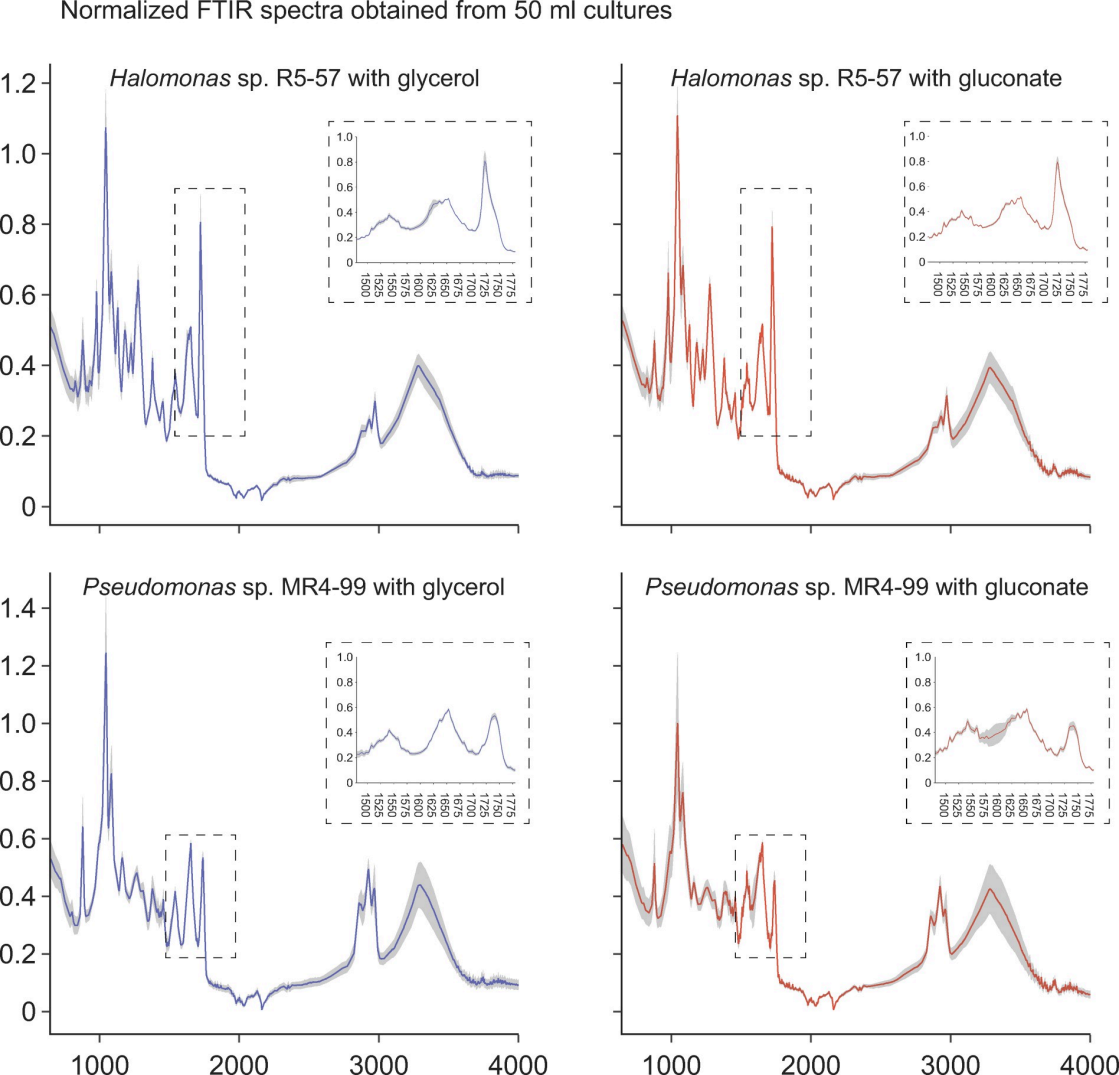
Normalized FTIR spectra obtained from 50 mL cultures. Cultures of *Halomonas* sp. R5-57 and *Pseudomonas* sp. MR4-99 were supplemented with glycerol or gluconate and cultivated for 120 and 72 hours, respectively (n = 3 for both strains). Cells were spun down, washed in ethanol and analyzed on a Cary-FTIR in technical triplicates. Carbonyl-ester peaks were visible similar to spectra obtained from the 150 μL cultures cultivated in 96-well plates. Production of PHA were verified for both strains by GC-FID analysis in the same samples from which the Cary-FTIR spectra were obtained.

The FTIR spectra obtained from 50 mL cultures (Fig 4) were consistent with the spectra obtained from 150 μL cultures in terms of containing carbonyl-ester peaks at the same strain specific wavenumbers. The intensity of carbonyl-ester peaks, and, thus the CA1 ratios, were however different. For *Halomonas* sp. R5-57, CA1 ratios close to 1.0 were found from the spectra obtained from 50 mL cultures (Table 2) while the CA1 ratios were 1.2 and 0.7 for the 150 μL cultures supplemented with gluconate and glycerol, respectively (S7 Fig). For *Pseudomonas* sp. MR4-99, a pattern of higher CA1 ratios for the 50 mL cultures was observed with CA1 ratios of approximately 0.4 (Table 2) compared with 0.3 for glycerol and 0.1 for gluconate in the 150 μL cultures (S8 Fig). This difference most likely reflects that the experimental conditions in terms of *e*.*g*. oxygen transfer rates (OTR) were different between the 150 μL and 50 mL cultures.

## Discussion

HTS methods have become widely adopted in research fields including microbial biotechnology, molecular biology, metabolic engineering, metagenomics, and others [41]. Despite the methodological development in related fields, only a few studies employed HTS approaches for screening or optimization of PHA production in bacteria. Such HTS methods are needed since production of microbial compounds are substrate dependent and usually tedious and labor intensive to screen for.

Our HTS approach was partly inspired by Smirnova *et al*. [16] who described cultivation of bacteria isolated from Antarctica in a 24-well deep plate Duetz Microtiter Plate System and analyzed for putative PHA production with the same HTS-XT FTIR system used by us. Other studies have used HTS to monitor bacterial growth in 96-well plates and quantified endpoint secretion of rhamnolipids by *Pseudomonas aeruginosa* [42], detected acetone, vanillin, and various ketones and aldehydes from *E*. *coli* supernatants in 96-well plates [43], and investigated the time-resolved metabolome profile of *Pseudomonas* spp. using serial dilution of the initial population by sampling at the same endpoint [44]. Combining different HTS methods with the use of the HTS-XT FTIR system might enable increased resolution of growth and PHA production dynamics in the future. The ideal HTS PHA screening setup could furthermore enable PHA screening of cells harvested directly from the Biolog plates, even if the low carbon to nitrogen ratios used in our experiments were too low to produce PHA. The exact concentrations of carbon substrates in the Biolog assay are undisclosed, but ranges from 2- to 20 mM according to the manufacturer. Thus, some carbon substrates will be present in a relatively low C:N ratio, and the ratio will differ greatly between substrates and make comparisons problematic. An experimental setup where the nitrogen source is supplied in different concentrations by use of several Biolog PM1 plates might allow for PHA production to be detected in cells cultivated under such conditions.

Several parameters such as type of carbon substrate and the OTR influences bacterial growth, morphology, and PHA production. Establishment of carbon substrate phenotypes are commonly performed as part of basic strain characterization [22] or to develop green bioprocesses using cheap substrates [43]. Determining the carbon substrate phenotype and the influence on bacterial morphology and PHA production is therefore highly relevant for PHA producing strains. It was previously shown for *Halomonas* sp. YLGW01 that supplementation with fructose resulted in production of 94% PHB and almost four times longer cells compared to use of glucose [25]. *Pseudomonas* strains isolated from the Arctic environment produce mcl-PHA from non-fatty acids such as glucose, fructose, and glycerol [26, 45], similar to demonstrated in this study for *Pseudomonas* sp. MR4-99 when supplemented with gluconate and glycerol.

Another influential parameter for bacterial growth, morphology, and PHA production is the OTR, which is lower in deep-well microtiter plates than in 250 mL shake flask cultures [46]. Although we used slightly different types of plates and shake flasks, a similar difference in OTR is to be expected between our small-scale- and shake flask cultures. Differences in OTR might help to explain observations of the PHA production measured *via* FTIR. When comparing CA1 ratios from Cary-FTIR spectra obtained from the 150 μL cultures of *Halomonas* sp. R5-57 with those from 50 mL cultures, a higher CA1 ratio was found in the small-scale cultures when *Halomonas* sp. R5-57 were supplemented with gluconate, while a lower CA1 ratio was obtained when glycerol was used. Although the Cary-FTIR data showed that the highest putative PHA production from small-scale cultures was substrate dependent, the higher CA1 ratio obtained for gluconate in the small-scale cultures compared to shake flask cultures could indicate that PHA production is higher when the OTR is low for this substrate. Such a correlation between low OTR and PHA production has previously been demonstrated in *Halomonas bluephagenesis* TD01 and explained by a mechanism where NADH rather than NADPH is used as co-factor for the acetoacetyl-CoA reductase PhaB in the PHA biosynthesis pathway [47].

On the other hand, fatty acid *de novo* synthesis of mcl-PHA such as in *Pseudomonas* spp. requires oxygen [48]. For *Pseudomonas* sp. MR4-99, the Cary-FTIR analyses did show a higher PHA production as indicated by higher CA1 ratios in the shake flask cultures compared to the small-scale cultures. This pattern is consistent with the higher reported OTR in shake flasks compared to 96-well plates. The low CDW obtained from 50 mL cultures furthermore suggest that the medium or substrate concentration used was sub-optimal for growth of *Pseudomonas* sp. MR4-99, or that the accumulation of mcl-PHA reduced the growth and thus the PHA production.

Previous analyses of FTIR spectra from *Rhodococcus yannanensis* G.S. 3 and *Polaromonas* sp. G. S. 42 obtained by the same HTS-XT FTIR system used in our study indicated production of PHA by the presence of carbonyl-ester peaks at 1741 cm^-1^ and 1738 cm^-1^, respectively [16]. This is highly similar to the 1739 cm^-1^ peak identified in the FTIR spectra of *Pseudomonas* sp. MR4-99. A lower peak wavelength was observed at 1723 cm^-1^ for *Halomonas* sp. R5-57. This difference in peak wavelength can be explained by production of mcl-PHA in *Pseudomonas* sp. MR4-99 versus scl-PHA in *Halomonas* sp. R5-57. Additional support for the PHA side chain configuration being responsible for the observed differences in peak wavelengths came from Cary-FTIR spectra from 50 mL and from 150 μL cultures of both strains, where carbonyl-ester peaks were observed from both types of cultures. The production of scl-PHA or mcl-PHA for *Halomonas* sp. R5-57 or *Pseudomonas* sp. MR4-99, respectively, was furthermore confirmed by GC-FID analyses of the 50 mL cultures. Taken together, the FTIR data suggest that PHA was produced by both strains when cultivated in the 96-well plates, thus enabling HTS screening of PHA production.

The Cary-FTIR spectra obtained from samples of the 50 mL cultures of *Halomonas* sp. R5-57 showed high CA1 ratios relative to *Pseudomonas* sp. MR4-99 when taken the lower amount of PHA produced into account (Table 2). Since FTIR analysis is still only an indirect method for PHA characterization in bacterial cells, other types of compounds and biopolymers, like rhamnolipids, can give rise to carbonyl-ester peaks at wavenumbers similar to PHA. Rhamnolipids are anionic biosurfactants and structurally related to PHA, as they contain a sugar moiety bound to a hydroxyalkanoic acid [21]. Carbonyl-ester peaks in FTIR spectra can therefore also originate from secreted rhamnolipids [49], if these are subsequently centrifuged with the cells. Neither *Halomonas* sp. R5-57 nor *Pseudomonas* sp. MR4-99 have been investigated for natural production of rhamnolipids, but cloning of the codon optimized rhamnolipid synthesis gene *rhlA* from *Halomonas* sp. R5-57 into *E*.*coli* resulted in production of rhamnolipid precursors [50]. Acylglycerides are another type of compounds that can show carbonyl-ester peaks in FTIR analyses, these are also secreted into the culture broth but typically produced in low quantities in bacteria [51, 52]. Strong carbonyl-ester peaks in FTIR spectra from bacteria are therefore generally more likely to originate due to accumulation of PHA.

Also, when evaluating or comparing CA1 ratios between different species of bacteria, the lower signal intensity of the single carbonyl-ester group relative to the higher molecular weight per molecule of mcl-PHA compared to scl-PHA should be considered as explanation for the lower CA1 ratios observed in FTIR spectra of *Pseudomonas* sp. MR4-99 compared to *Halomonas* sp. R5-57. This combined with results from other studies using FTIR suggest that differences in morphology and chemical composition of bacteria and bacterial polymers means any attempt to make quantitative models for PHA should be species- or strain- and instrument specific [53–55].

Despite FTIR being only indicative and semi-quantitative for PHA production in bacteria, the presented methodology could be of interest to find optimized multivariate conditions for PHA production such as vitamin supplementation, salt requirement, nitrogen substrates, or changes in pH. When combined with high-throughput FTIR analysis, time-resolved data can also potentially help elucidate the effect of PHA production on population growth dynamics, of which not much data is currently available. Furthermore, FTIR spectra can be calibrated to the reference GC-FID data with the subsequent development of prediction models by applying advanced multivariate data analysis approaches [56–60], as it was successfully done for acyl glycerides and free fatty acids [61–63]. This would enable use of FTIR spectroscopy as quantitative methodology replacing tedious GC-FID analysis. Finally, FTIR spectroscopy combined with the recently developed automated sample preparation solutions [64–66] allow to perform rapid and extraordinary HTS that would bring optimization of PHA production to the advanced level of complexity.

## Conclusion

In this study, the marine bacteria *Halomonas* sp. R5-57 and *Pseudomonas* sp. MR4-99 were tested in a HTS setup for PHA production *via* FTIR analysis. The strains were characterized for growth on 95 different carbon substrates using the Biolog PM1 96-well plate system. Fifteen (*Halomonas* sp. R5-57) and 14 (*Pseudomonas* sp. MR4-99) substrates were selected for further analyses of the two strains in 96-well plates, and the “maximum specific growth rate” and “area under the curve” were calculated using the Python growthcurver tool AMiGA. FTIR analyses of the cells harvested at the end of the 96-well plate experiments indicated production of scl-PHA (*Halomonas* sp. R5-57) and mcl-PHA (*Pseudomonas* sp. MR4-99). PHA production was finally confirmed in 50 mL cultures for both strains when supplemented with glycerol or gluconate. The FTIR spectra obtained from 150 μL and 50 mL cultures showed similar characteristic carbonyl-ester peak wavenumbers. Thus, this study confirms that PHA can be produced in cells cultivated in small volumes as 96-well plates, thereby enabling HTS of bacterial PHA production using FTIR. Our study results are a starting point for the establishment and standardization of HTS for PHA production by bacteria, which can aid in strain characterization and optimization experiments and thus help to pave the way for industrial production of PHA bioplastics, which is, among other factors, supported by the use of green and cheap carbon substrates.

## Supporting information

S1 Fig Biolog PM1 absorbance measurements for *Halomonas* sp. R5-57. (HTML)

S2 Fig Biolog PM1 absorbance measurements for *Pseudomonas* sp. MR4-99. (HTML)

S3 Fig Normalized Cary-FTIR spectra obtained from 150 μl cultures of *Halomonas* sp. R5-57. (HTML)

S4 Fig Normalized Cary-FTIR spectra obtained from 150 μl cultures of *Pseudomonas* sp. MR4-99. (HTML)

S5 Fig Normalized HTS-XT-FTIR spectra obtained from 150 μl cultures of *Halomonas* sp. R5-57. (HTML)

S6 Fig Normalized HTS-XT-FTIR spectra obtained from 150 μl cultures of *Pseudomonas* sp. MR4-99. (HTML)

S7 Fig The carbonyl- to amide band I ratios calculated from Cary-FTIR spectra obtained from 150 μl cultures of *Halomonas* sp. R5-57. (HTML)

S8 Fig The carbonyl- to amide band I ratios calculated from Cary-FTIR spectra obtained from 150 μl cultures of *Pseudomonas* sp. MR4-99. (HTML)

## Abbreviations

Abs_590nm_: Absorbance at wavelength 590 nm
ATR: Attenuated Total Reflectance
AU: Arbitrary units
AUC: Area under the curve
C:A: Carbonyl-ester to amide band I-ester ratio
C:N: Carbon to nitrogen ratio
CDW: Cell dry weight
FTIR: Fourier transform infrared spectroscopy
GC-FID: Gas Chromatography-Flame Ionization Detector
HTS: High-throughput screening
LB: Lysogeny broth
KEGG: Kyoto Encyclopedia of Genes and Genomes
NADH: Nicotinamide adenine dinucleotide
MA: Marine agar
MP: Marine peptone
PHA: Polyhydroxyalkanoate(s)
PHB: Polyhydroxybutyrate
PHBV: Polyhydroxybutyrate-co-valerate
PHH: Polyhydroxyhexanoate
PHO: Polyhydroxyoctanoate
PHD: Polyhydroxydecanoate
OD_600nm_: Optical density at wavelength 600 nm
OTR: Oxygen transfer rate
YE: Yeast extract
